# Critical Thinking: Creating Job-Proof Skills for the Future of Work

**DOI:** 10.3390/jintelligence11100194

**Published:** 2023-10-09

**Authors:** Daniela Dumitru, Diane F. Halpern

**Affiliations:** 1 Teacher Training Department, Bucharest University of Economic Studies, 010374 Bucharest, Romania; 2 Doctoral School of Psychology and Educational Sciences, University of Bucharest, 050663 Bucharest, Romania; 3 Department of Psychology, Claremont McKenna College, Claremont, CA 91711, USA; dianefhalpern@gmail.com

**Keywords:** critical thinking, artificial intelligence, job market, job disruption

## Abstract

In this study, we explore the transformative impact of artificial intelligence (AI) on the job market and argue for the growing importance of critical thinking skills in the face of job automation and changing work dynamics. Advancements in AI have the potential to disrupt various professions, including, for example, programming, legal work, and radiology. However, solely relying on AI systems can lead to errors and misjudgments, emphasizing the need for human oversight. The concept of “job-proof skills” is introduced, highlighting the importance of critical thinking, problem-solving, empathy, ethics, and other human attributes that machines cannot replicate with the same standards and agility. We maintain that critical thinking can be taught and learned through appropriate classroom instruction and transfer-focused approaches. The need for critical thinking skills is further reinforced by the influx of information and the spread of misinformation in the age of social media. Moreover, employers increasingly value critical thinking skills in their workforce, yet there exists a gap between the demand for these skills and the preparedness of college graduates. Critical thinking is not only essential for the future of work, but also for informed citizenship in an increasingly complex world. The potential impact of AI on job disruption, wages, and employment polarization is discussed, highlighting the correlation between jobs requiring critical thinking skills and their resistance to automation. We conclude by discussing collaborative efforts between universities and labor market organizations to adapt curricula and promote the development of critical thinking skills, drawing on examples from European initiatives. The need to prioritize critical thinking skills in education and address the evolving demands of the labor market is emphasized as a crucial step for navigating the future of work and opportunities for workers.

## 1. Introduction: Critical Thinking: Creating Job-Proof Skills for the Future of Work

The rapid evolution of online technologies has ushered in a paradigm shift in employment, redefining the nature of work and the skills required to succeed in the digital age. This transformative landscape, characterized by the ubiquitous presence of the Internet, social media platforms, and advanced artificial intelligence systems, has created a plethora of new opportunities and challenges in the labor market. As we navigate this digital frontier, it is becoming increasingly clear that traditional employment paradigms are undergoing a profound transformation. The convergence of online technologies with the demands of a networked world has not only created new job opportunities, but it has also disrupted established industries, rendering some job roles obsolete while creating demand for previously unforeseen skills. In this era of unprecedented connectivity and innovation, examining the intricate interplay between online technologies and jobs is paramount as it holds the key to understanding the dynamics of our rapidly evolving workforce.

Artificial intelligence (AI) is disrupting many jobs and promises “to change the way the world works” (adminGPT 2023, para. 13). The number and range of AI programs are increasing at a rapid pace, and they are likely to continually improve to meet user demands. Consider, for example, ChatGPT, which can respond to questions and requests in a way that seems to come from a human rather than a computer program. GPT stands for “generative pretrained transformer”. It is generative in that it can provide responses that it never “learned”; it is pretrained with a large language model (Bushwick et al. 2023). Newer versions can describe visual images, although thus far, they cannot create visual images. Its uses are seemingly endless. It is easy to imagine how such programs can change the lives of blind individuals. In fact, it can and will change the lives of all of us.

In this paper, we argue that these advances in online technologies will make critical thinking (CT) more important than ever before. Many who are preparing to enter the job market, and many who are already employed, will need to adapt to new forms of job automation and different ways of working. 

Consider, for example, that an early achievement of ChatGPT was its generation of Python code (a computer language) to compute various tasks, such as data analysis. Apparently, getting ChatGPT to generate code is so easy that several YouTube videos have popped up claiming that they can teach novice users to use ChatGPT to generate code in 90 s. (Data Professor 2023). The benefits are obvious, but so are the potential job losses for people who work in Python. Python coders will need to upgrade their skills, perhaps first becoming experts in the use of ChatGPT and similar programs, but this also has a positive side--they can spend more time working on larger questions such as which analyses are needed, and, of course, carefully reviewing the work produced by AI to ensure that it is accurate and understandable. Early versions of ChatGPT responses often contained errors. A New York lawyer learned the hard way: Steven A. Schwartz, a lawyer for 30 years, used ChatGPT to create a legal document (Weiser and Schweber 2023). It was filled with fake citations and bogus judicial opinions. Sadly, Mr. Schwartz never checked the accuracy of the document he filed in court. The judge was not amused. This highly public and embarrassing event should be a lesson for all of us. Current AI programs cannot be trusted to take over our work, though they may be able to aid or supplement it. However, other AI programs can “read” radiographs more accurately than human radiologists, which provides a benefit to both radiologists and patients. There is an immediate positive effect for this advancement: Radiologists will have more time to directly work with patients, and yes, they must also check the accuracy of the outputs from their programs when presenting diagnoses. 

For the rest of us, whether we are students or early or late in our careers, we need to focus on the development of “job-proof skills” in the face of AI advances. A report from the United Nations defines job-proof skills as “conceptual and strategic thinking, problem-solving, empathy, optimism, ethics, emotional intelligence, and judgments are the future-proof skills and attributes that machines will not be able to replicate with the same standards and agility as qualified human beings” (Elkeiy 2022, para. 5). In other words, critical thinking skills will always be needed. 

## 2. What Is Critical Thinking?

Although some scholars in the field of critical thinking have emphasized differences among various definitions, we believe that the commonalities are evident (c.f., Dwyer 2017; Nisbett 2015; Lipman 1991; Fisher 2001). There are some differences in the use of terms and several skills might be more important, but all of the definitions (more or less) conform to our preferred definition: “Critical thinking is the use of those cognitive skills and abilities that increase the probability of a desirable outcome. It is purposeful, reasoned, and goal directed. It is the kind of thinking involved in solving problems, formulating inferences, calculating likelihoods, and making decisions. Critical thinkers use these skills appropriately, without prompting, and usually with conscious intent, in a variety of settings. That is, they are predisposed to think critically. When we think critically, we are evaluating the outcomes of our thought processes--how good a decision is or how well a problem is solved. Critical thinking also involves evaluating the thinking process--the reasoning that went into the conclusion we’ve arrived at, or the kinds of factors considered in making a decision” (Halpern and Dunn 2023, pp. 6–7). The reason we need a common definition of critical thinking is that, without it, instructors can and have passed almost anything off as instruction in critical thinking. However, common ground is to be found concerning CT definitions. In a European project, which we shall refer to in Section 4.3, the critical thinking definition is based on the works of Halpern and Dunn (2023), Facione (1990), Paul and Elder (2008), and Kuhn (1999). During two debate sessions, 33 international participants from higher education and the labor market defined critical thinking as a deliberate cognitive process guided by conscious, dynamic, self-directed, self-monitored, and self-correcting thought (Rebelo et al. 2023). It relies on both disciplinary and procedural knowledge, along with metacognitive aspects (including metacognitive, meta-strategic, and epistemological dimensions). Critical thinking can be cultivated and enhanced through the development of competencies, and it is facilitated by various attitudes, such as systematic thinking, open-mindedness, empathy, flexibility, and cognitive maturity. Additionally, it encompasses intellectual skills such as reflection, self-regulation, analysis, inference, explanation, synthesis, and systematic thought. Critical thinking not only stimulates problem-solving capabilities but also facilitates effective communication, fosters independent and holistic thinking, and bolsters decision-making and active citizenship (Pnevmatikos et al. 2021). 

### 2.1. Can Critical Thinking Be Learned?

We teach writing, oral communication, and mathematics with the (often implicit) belief that these skills will be learned and transferred to multiple settings both inside and outside of the classroom. There is a large and growing research literature showing that, with appropriate classroom instruction in critical thinking, including specific instruction designed for transfer, the skills will spontaneously transfer and in uncued (i.e., there are no reminders to use the critical thinking skill that was learned in class) situations (Dumitru 2012; Heijltjes et al. 2014; Tiruneh 2019). Several such studies were presented by Dwyer (2017) and Halpern and Dunn (2023). For the sake of brevity, we review just one recent study. The study was designed to counteract the effects of conspiracy theories. When people believe conspiracy theories, they often act in harmful ways–such as refusing to get the COVID-19 vaccine, which resulted in the death of large numbers of people around the world, or attacking the United State Capitol Building on 6 January 2021 in the belief that there was a conspiracy afoot designed to steal the United States 2020 presidential election from Donald Trump. In a review of the research literature on the efficacy of interventions, the researchers found “there was one intervention which was characteristically different to the rest” (O’Mahony et al. 2023, para. 23). It was a semester-long university course in critical thinking that was designed to teach students the difference between good scientific practices and pseudoscience. These courses require effort and commitment, but they are effective. The same conclusion applies to all interventions designed to enhance critical thinking. There are no fast and easy “once and done” strategies that work. This is why we recommend continuous and pervasive coursework to make sure that the learning of CT skills “sticks.” 

### 2.2. The Need for Critical Thinking Skills

Online technologies-related (including AI) job loss and redesign are not the only reasons why we need to concentrate on teaching and learning the skills of critical thinking. COVID-19 left 140 million people out of work, and many of their jobs will never return (Roslansky 2021). We are drowning in a tsunami of information, confronted with advertisements online, in news reports, social media, podcasts, and more. The need to be able to distinguish good information from bad is critical. In addition, employers want to hire people with critical thinking skills. In a recent report by Hart Research Associated (2018), they found that in an employer survey of 501 business executives, 78% said that critical thinking/analytic reasoning is the most important skill they want in their employees, but they also added that only 34% of college graduates arrive well prepared in critical thinking. This gap between what employers want and their perception of the preparedness of the workforce was larger for critical thinking than for any other area. In fact, every report on the future of work made this same point. Consider this quote from The World Economic Forum (2020) on the future of jobs: “Skills gaps continue to be high as in-demand skills across jobs change in the next five years. The top skills and skill groups which employers see as rising in prominence in the lead up to 2025 include groups such as critical thinking and analysis as well as problem-solving.” (p. 5). In a report from the Office of the European Union: Key Competences for Lifelong Learning, the commissioner wrote “Critical thinking, media literacy, and communication skills are some of the requirements to navigate our increasingly complex world” (Navracsics 2019, p. 3). Of course, critical thinking is not just needed in the world of work. A true democracy requires an educated citizenry with citizens who can think critically about world social issues, such as the use/threat of AI, war, poverty, climate change, and so much more. Irrational voters are a threat to all of us—and to themselves. 

The need to think critically is not new, but it has taken on a new urgency as social media and other forms of communication have made the deliberate spread of misinformation move at the speed of light. There is nothing new about the use of lies, half-truths, and innuendos to get people to believe something that is not true. Anyone can post anything on popular media sites, and this “fake news” is often copied and shared thousands of times. Sometimes the information is spread with a deliberate attempt to mislead; other times, it is copied and spread by people who believe it is true. These messages are often used to discredit political adversaries, create social unrest, and incite fear. It can be a difficult task to determine what to believe and what to discard. Vosoughi et al. (2018) analyzed data from 126,000 tweets that were spread by approximately 3 million people. How did the researchers discriminate true data from false data? The same way we all should. They used several different fact-checking sites and found 95% to 98% agreement regarding the truth or falsehood of information. They found that false data spread more quickly and more widely than true data because the false data tended to be novel and sensational, rendering it salient and seductive. 

In today’s landscape, the imperative to foster critical thinking skills is becoming increasingly apparent as we grapple with the rapid rise of social media and artificial intelligence technologies and their profound impact on the future of work. The confluence of these transformative forces has ushered in a new era characterized by the potential for significant job disruption. As online technologies advance and automation becomes more widespread, certain traditional job roles may become obsolete, requiring the development of innovative skills and adaptability in the workforce. In this context, critical thinking emerges as a central element in preparing individuals to navigate the evolving job market. It equips individuals with the ability to analyze complex information, discern credible sources from the proliferation of social media information, and make informed decisions in an era of blurring boundaries between human and machine contributions to the workforce. Cultivating critical thinking skills will be essential to ensuring that individuals can take advantage of the opportunities presented by new technologies while mitigating the challenges of job disruption in this AI-driven future.

## 3. Critical Thinking Skills and Job Disruption and Replacement

Eloundou et al. in 2023 estimated that about 15% of all U.S. workers’ jobs could be accomplished much faster and at the same level of quality with currently available AI. There are large differences in the extent to which various occupations and industries will be affected by advancements in AI. For example, tasks that require a high degree of human interaction, highly specialized domain knowledge, or creating innovative technologies will be minimally affected; whereas, other occupations such as providing captions for images or answering questions about a text or document are more likely to be affected. Routine-based jobs in general are more likely to be dislodged by advanced technologies (Acemoglu 2002). Using the basic definitions of skills that are standard in O*Net, Eloundou et al. (2023) found a clear negative correlation between jobs requiring knowledge of science and critical thinking skills and the likelihood that AI will “take over” the job. These findings reinforce our main point—the best way to gain job-proof skills is with critical thinking. 

The effect of online technologies on wages is complicated because of the large number of factors that come together to determine earnings. Acemoglu and Autor (2011) advocated for a model that simultaneously considers the level of the tasks required for any job (low, medium, and high), where a high level of skill is defined as one that allows employees to perform a variety of tasks, the demand for the tasks, and technological changes that can complement a task or replace it. They assert that employment has become increasingly polarized with the growth in both high education, high wage occupations and low education, and low wage occupations in the United States and the European Union. To understand and predict which occupations will be most disrupted by AI (and other developing technologies), an investigator will need to simultaneously consider all of these variables. Technological advancements can generate shifts in demand, favoring either high- or low-skilled workers. According to Acemoglu and Autor (2011), we can expect some of the largest disruptive effects at the middle level of skills, where some of the tasks performed at this level can be more easily replaced by new technologies, with widespread employment growth in high- and low-skilled occupations.

## 4. Business-University Collaborations 

The pursuit of promoting high standards of critical thinking in university students across various academic disciplines is a challenging endeavor that should be leveraged through collaboration with stakeholders. In such collaborations, stakeholders can contribute to refining the skills required by learners and bring their own perspectives to academic instruction. This close partnership between universities and stakeholders helps minimize gaps and mismatches in the transition to the labor market, facilitates research collaboration, and increases student motivation.

Collaborations between businesses and universities have gained increasing importance in today’s rapidly evolving educational and economic landscape. These partnerships are instrumental in bridging the gap between academic learning and the real-world skills demanded by the job market. One key aspect of business-university collaboration (BUC) is the alignment of curricula with the dynamic needs of industries. This entails the joint effort of higher education institutions (HEIs) and industry experts to design, develop, and deliver educational programs that equip students with practical, job-ready skills. The curriculum design phase involves tailoring study programs, courses, and modules to address skills gaps and align with the specific requirements of employers.

Moreover, BUC extends beyond the classroom. Collaborations often involve business engagement in educational activities, including guest lectures, internships, co-op programs, and research projects. These interactions provide students with invaluable exposure to real-world scenarios, allowing them to apply theoretical knowledge in practical settings.

In essence, BUC is a multifaceted partnership that benefits both students and businesses. It ensures that educational programs remain relevant, fostering a seamless transition from academia to the workforce. This collaborative approach not only enhances students’ employability but also contributes to the overall growth and innovation of industries.

Operationalizing the collaboration implicates a particular focus on curriculum design, development, and delivery. These involve the collaboration between higher education institutions and labor market partners to create or enhance undergraduate or postgraduate study programs, courses, or modules. This collaborative effort aims to address skills gaps, align curricula with employers’ needs, integrate training initiatives, and improve graduates’ employability. Additionally, curriculum delivery includes various forms of business involvement, such as guest lectures, placements, supervision, mentoring, and work-based learning activities.

While the existing literature often discusses the barriers and motivations for university-business collaboration (Healy et al. 2014; Orazbayeva et al. 2020), there is a need for more empirical insights into the roles and responsibilities of each party engaged in joint curriculum design, development, and delivery, as well as lessons learned from these collaborations (Rebelo et al. 2023).

### 4.1. Why Do We Need Higher Education’s Help?

In the preceding sections of this paper, we delved into the disruptive forces of artificial intelligence (AI) on the job market and the critical need for individuals to adapt to these changes by developing “job-proof skills”. The rise of online technologies such as ChatGPT presents both opportunities and challenges, particularly in fields where middle-level skills are required. To effectively tackle these challenges, we must turn our attention to the pivotal role of education and the cultivation of essential skills such as critical thinking.

We highlighted how AI is rapidly transforming various industries and the need for individuals to adapt to these changes. Moreover, we explored the question of whether critical thinking can be learned, showcasing research evidence that supports the teachability of this skill. Now, we shall explore practical strategies for fostering critical thinking skills through collaborations between universities and businesses. The idea here is to create an educational framework that equips students with the capabilities needed to thrive in the evolving workforce.

Building upon the success of two European projects, “Critical thinking across higher education curricula—CRITHINKEDU” and “Critical thinking for successful jobs—THINK4JOBS”, we argue that incorporating practical experience and CT development through apprenticeships is a possible action for better higher education classes. This collaborative approach between HEI and LMO designed to address the differing perspectives and terminologies used by these two entities regarding critical thinking could be an important curriculum design for the better adaptation of job market technology disruptions.

Research conducted by Eloundou et al. (2023), which shows that critical thinking skills and science skills are less likely to be taken by AI, compels us to sustain the THINK4JOBS apprenticeship curricula as a possible teaching protocol for critical thinking enhancement to face challenges posed by AI at work.

The results from these projects demonstrate significant progress in students’ critical thinking skills and dispositions. These improvements, as highlighted below in Section 4.3, underscore the effectiveness of embedding critical thinking in the curriculum. The guidelines formulated for implementing Critical Thinking Blended Apprenticeship Curricula provide a roadmap for educators to follow when effectively integrating critical thinking into their courses.

As we ponder the possibility of a world where critical thinking is widespread, we can envision a future where individuals are equipped to confront the ideological fanaticism that threatens global stability. Critical thinking, as both a cognitive skill and a disposition, has the potential to shape a workforce capable of adapting to the ever-changing landscape of work, making informed decisions, and contributing to a more rational and democratic world. The THINK4JOBS project emphasizes the practical steps taken to prepare students for the future job market and sets the stage for further exploration of the role of critical thinking in addressing global challenges, including AI presence in the job market. 

### 4.2. CRITHINKEDU Proctocol for Critical Thinking Education across Curricula

Given that the best education for the future of work is the acquisition of critical thinking skills, how can we facilitate this sort of education? One way to obtain a job-proof education is to create classes with the help of labor market organizations. Two projects funded by the European Union were designed to bring to life the idea that better communication and collaboration between universities and employers result in a better adaptation of the curriculum, especially a curriculum involving critical thinking skill development. 

Between 2016 and 2019, the project “Critical thinking across the European higher education curriculum—CRITHINKEDU” focused on how CT is taught in various academic domains. The CRITHINKEDU project, involving universities across Europe, exemplifies how academia and industry can join forces to bridge the gap between classroom learning and real-world job demands. This initiative aimed to enhance the curriculum by explicitly emphasizing critical thinking skill development. It revealed that employers across various fields value critical thinking, and they perceive it as essential for recent graduates entering the workforce.

The participants were eleven universities from nine European countries (Belgium, Czech Republic, Greece, Italy, Spain, Portugal, Romania, Lithuania, and Ireland; Dominguez 2018). Qualitative research was conducted with 32 focus groups comprised of professionals from various European countries and fields. The findings align with previous studies: “CT is a set of interconnected skills (interpretation, inference, analysis, explanation, evaluation, self-regulation”, see Payan-Carreira et al. (2023, p. 16), and dispositions (open-mindedness, refection, attentiveness, organization, perseverance, intrinsic goal motivation (Payan-Carreira et al. 2023), essential for recent graduates in response to labor market demands. However, an important consideration is that the practical application of CT varies across professional fields. The participants in this study defined the ideal critical thinker as someone with a cultivated mindset, motivated to learn and improve, and equipped with cognitive and behavioral tools to anticipate, regulate, and monitor their thinking. CT is associated with problem-solving and decision-making and is intertwined with other skills such as proactivity, adaptability, creativity, emotional intelligence, communication, and teamwork. The report from this project also introduced “a European collection of the Critical Thinking skills and dispositions needed in different professional fields for the 21st century” (Dominguez 2018), which categorizes CT skills and dispositions based on professional fields and offers a basis for defining learning objectives and adapting university curricula. This study provides valuable insights from 189 European employers into CT needs in the labor market for new graduates. The interviewed professionals had an obvious preference for CT skills in STEM fields and an obvious preference for dispositions in the Humanities. Social Sciences and bio-medical sciences professionals were equally interested in CT skills and dispositions, with a slight preference for dispositions (Dominguez 2018, p. 28).

### 4.3. Next Steps: THINK4JOBS Blended Appreticeship Curricula 

After the termination of the CRITHINKEDU project, partners from Romania, Greece, Lithuania, and Portugal, with the addition of a new partner from Germany, proposed a new research application: “Critical Thinking for Successful Jobs—THINK4JOBS” (www.think4jobs.uowm.gr). The idea was to utilize the results from the previous project and, together with labor market organizations, create new courses that are more adapted to the reality of the future of work. The core element of the classes was explicit teaching of critical thinking, using real-life cases and methods. In an apprenticeship model, critical thinking skills are embedded in a relevant context. The value of realistic contexts is that students can see the need for the skills being taught in a workplace scenario. Relevant contexts enhance student engagement and motivation to learn. Dumitru et al. (2021) focused on improving students’ critical thinking skills and dispositions through collaboration between Higher Education Institutions (HEIs) and Labor Market Organizations (LMOs). The aim was to bridge the gap between HEI curricula and the expectations of the labor market by incorporating apprenticeships that provide practical experience and CT development.

The process of mapping responses from those in the labor market organizations onto college curricula involved the use of research methods such as observation, focus groups, and documentary analysis, with stakeholders from HEIs and LMOs participating. The findings indicated that while there were no definitive “gaps” between HEIs and LMOs, there were contextual differences in the approach to CT. HEIs focus on long-term career preparation, while LMOs emphasize short-term learning strategies. The terminology and expression of CT also differed between the two contexts. Based on the findings, ten work-based scenarios were created, with one from each discipline involved in the project. Overall, the report (Dumitru et al. 2021) highlighted the different goals and perspectives of HEIs and LMOs regarding CT, emphasizing the need for collaboration and a common understanding of which skills should be included in the college curriculum.

There is a different context in the approach to CT, since HEIs usually use different learning activities, focusing more on career preparation with long-term goals, while LMOs follow compact and short-term learning and teaching strategies. Furthermore, the findings suggest that CT is a new workplace requirement and that HEIs and LMOs do not choose the same terminology when referring to the concept, with HEIs usually choosing scientific terms. Another element that emerged is that CT is generally expressed in a declarative way in higher education institutions, while in LMOs the application to specific cases follows a more procedural approach. Put another way, LMOs are focused on making a profit, while HEI is focused on being socially responsible. 

In the second phase of the project, partners (Pnevmatikos et al. 2021) focused on the development of a collaborative training curriculum for Higher Education Instructors and LMO tutors. The purpose of the training was to enhance comprehension and knowledge of critical thinking for both sides of this collaboration, since previous research indicated a potential lack of conceptual and procedural understanding between these two entities. Additionally, the training aimed to facilitate the promotion, support, and evaluation of students’ CT skills within apprenticeship curricula, as well as the creation of blended curricula utilizing an open-source learning platform. The training course encompassed workshops that delved into various aspects of CT, including analyzing and reassembling ideas about CT, formulating a working definition of CT, instructional methodologies, blended learning techniques, usage of a learning platform, CT assessment, and the development of a Memorandum of Understanding (MoU) between higher education institutions and LMOs. The participants’ knowledge about these topics was assessed through pre- and post-training online questionnaires. Although data analysis showed various predicted trends, only perceived self-confidence in the topics covered during the training obtained statistical significance (Pnevmatikos et al. 2021).

In the final report from this project, Payan-Carreira et al. (2023) presented the results of the implementation of the critical thinking Blended Apprenticeships Curricula (CTBAC) and discussed the improvements in critical thinking skills and dispositions observed in students. The study involved cross-disciplinary analysis and assessed changes before and after the piloting activities. A total of 609 students participated, and their critical thinking skills and dispositions were evaluated. 

The consortium chose the Critical Thinking Self-Assessment Scale (CTSAS) developed by Nair (2011) as an instrument to assess CT skills based on an earlier conceptualization (Facione 1990). The questionnaire has been tested in various geographic and cultural contexts, demonstrating good reliability, internal consistency, and confirmatory factor analysis results. However, the original CTSAS was considered too long to complete, consisting of 115 items, so a shorter version was specifically developed for this project. The short form of the questionnaire (CTSAS-SF) was created through a two-step process. Items with loading weights below .500 were eliminated, resulting in 84 remaining items. Redundant and non-cognitive-focused items were marked for elimination, leaving 60 items. The short form maintained the original scale’s framework and utilized a seven-point Likert scale ranging from 0 (Never) to 6 (Always) for students to respond to items assessing various dimensions and subdimensions of CT skills.

The CTSAS-SF validation process, with confirmatory factor analysis, resulted in two models with equivalent satisfactory goodness-of-fit indices. Model 4, the second-order factor model (RMSEA = .051; TLI = .924; CFI = .927), had a chi-square/df ratio of 2.33. The Cronbach alpha of the overall instrument was excellent (α = .969). Sample items are shown in Table 1.

Compared to instruments for assessing CT skills, the availability of instruments for measuring critical thinking (CT) dispositions is limited. However, one of the instruments adopted by the consortium to assess CT dispositions is the Student-Educator Negotiated Critical Thinking Dispositions Scale (SENCTDS), which was developed by Quinn et al. (2020). The scale was validated with a mixed population of Irish and American undergraduate students. The scale considers a variety of CT dispositions that the authors consider important for the labor market and real-world decision-making. Some of the items in the scale combine Facione’s (1990) original CT dispositions into new dimensions that are relevant to academic and labor market success, such as organization, perseverance, and intrinsic goal motivation. The scale consists of six dimensions (Reflection, Attentiveness, Open-mindedness, Organization, Perseverance, and Intrinsic Goal Motivation) and presents statements for students to respond to using a 7-point Likert scale. The Likert scale ranges from 1 (strongly disagree) to 7 (strongly agree). The original version of the SENCTDS contains 21 items. The validation process, with confirmatory factor analysis, identified only one model presenting a satisfactory goodness-of-fit index—model 3, comprised of six correlated factors (RMSEA = .054; TLI = .974; CFI = .969) with a chi-square/df ratio of 2.57. The instrument presented a high Cronbach alpha (α = .842), suggesting a strong internal consistency of the instrument. Sample items are presented in Table 2. 

The analysis showed gains in critical thinking skills and indicated that changes were more prominent in skills than dispositions. All skills (interpretation, analysis, inference, explanation, self-regulation, and evaluation) obtained significant differences between the pretest and posttest, with *p* ≤ .0001 to all skills, plus the integrated critical thinking skills score was t = 9.705 and *p* ≤ .0001, which demonstrates strong significant difference between pre- and the posttest. Dispositions displayed no significant differences regarding the integrated score, but showed significant differences in reflection (t = 1.766, *p* = .079), open-mindedness (t = 2.636, *p* = .009), organization (t = 2.568, *p* = .011), and intrinsic goal motivation (t = 1.712, *p* = .088).

Based on the findings from the implementation of the blended apprenticeship curricula, the following guidelines were formulated for implementing Critical Thinking Blended Apprenticeship Curricula (Payan-Carreira et al. 2023):

- Provide an explanation of the importance of critical thinking—Clearly communicate to students why critical thinking is a vital skill in today’s workforce and how it is valued in specific professions. Explicitly incorporate the development of critical thinking as an outcome of the course.
- Emphasize continuous and pervasive CT training—To achieve success, there should be a concerted effort across disciplinary curricula to foster students’ critical thinking skills and dispositions. Skills require training, and dispositions necessitate the internalization of desired attitudes. Therefore, sufficient time and a collaborative approach at the disciplinary level are necessary for consistent and significant progress.
- Allocate dedicated time—Building on the previous point, it is essential to allocate specific time within the course to work on the proposed critical thinking goals. Students and educators need to schedule activities and create opportunities for preparation, development, and feedback exchange. This ensures that the intervention leads to meaningful, lasting learning.
- Establish connections with real-world scenarios—Foster student engagement and improve their perception of learning experiences by incorporating case studies that reflect situations professionals encounter in their daily work. By grounding the learning content in reality, students are more likely to be motivated and actively participate in the educational process.

Foster reflection on CT skills and dispositions—Offer students the chance to reflect on their reasoning processes and the attitudes they have developed throughout their learning experiences. Encouraging reflective thinking enhances the effectiveness of learning interventions and helps cultivate a deeper understanding of one’s experiences.

These steps aim to guide educators in effectively implementing the critical thinking blended apprenticeship curricula while also maximizing the impact of critical thinking development in students.

The two European projects made a great start in integrating the skills that employers want employees to learn from university curricula, but the results are nonetheless provisional. There is not a clear agreement among participating universities regarding how best to teach critical thinking, nor any regarding its importance for future jobs. We urge that more work should be done to nurture critical thinking within university curricula in order to provide our current students—who represent the future of the workforce—the much-wanted job-proof skills they need.

## 5. European Recommendations and Good Practices

Critical thinking stands as a pivotal goal for European Higher Education Institutions. To facilitate the attainment of this objective, we present an educational protocol that draws from comprehensive research and practical experiences, including insights from the CRITHINKEDU project. This protocol amalgamates insights from both theoretical and empirical studies on critical thinking with practical strategies for its cultivation.

Recommendations go toward signing memorandums of understanding between universities and labor market organizations to cultivate strong partnerships (Rebelo et al. 2023). Effective collaboration between universities and businesses is crucial in fostering critical thinking. This partnership thrives on the synergy that results when academic institutions and businesses combine their expertise, resources, and perspectives. Strategies such as aligning goals, fostering long-term commitment, and promoting a culture of collaboration can strengthen these partnerships and ensure that academic research is harmoniously aligned with real-world needs.

Another recommendation relates to the *formulation of compelling goals*. Accurate and transparent goals are fundamental to the successful implementation of university-industry collaborations to promote critical thinking. These goals must be clearly defined and easily understood at multiple levels, from the institutional to the program and course levels. Recognition of critical thinking as an overarching goal implies its integration into assessment and evaluation processes.

Another recommendation is to *develop flexible curricula*. To effectively foster critical thinking, curricula must demonstrate adaptability and responsiveness to emerging trends and market demands. The use of agile curriculum design methodologies and the involvement of business partners in curriculum development is of great value. Approaches such as problem-based and case-based learning facilitate rapid adaptation to evolving market needs, such as the use of AI-powered software to solve work tasks better and faster. Regular feedback mechanisms and ongoing collaboration with business partners ensure that curricula remain relevant and flexible.

*Incorporating real-world challenges and case studies into curricula* bridges the gap between academia and the business world, creating an environment that encourages experiential learning. The active involvement of business stakeholders in providing relevant challenges plays a key role. Students’ problem-solving skills are enhanced by shifting from traditional teaching methods to project-based, problem-based, or case-based learning. Engaging students through apprenticeships, internships, guest lectures, and seminars immerses them in authentic work environments and fosters their professional development.

Ongoing, multi-faceted evaluation is a cornerstone of the collaboration between higher education and the business community to cultivate critical thinking. Assessment includes measuring learners’ progress in critical thinking, the effectiveness of curricula, and the impact of partnerships through the use of key performance indicators. 

Regarding how to implement a critical thinking curriculum, pedagogical research (Elen et al. 2019) suggests that in the development of critical thinking, whether it is regarded as a skill, disposition, or a combination of both, three categories of supportive measures can be identified: modeling, induction, and declaration. 

Modeling: Support the development of critical thinking skills by demonstrating what it means to think critically at the institutional, programmatic, and course levels, considering multiple perspectives and alternative viewpoints.

Induction: Support critical thinking development by provoking critical thinking through the presentation of open-ended questions, unstructured tasks, complex problems, and real-world issues. The exact nature of “induction” and how it is implemented may vary across fields and disciplines. Induction can be carried out in a variety of ways; for example, presenting unstructured problems, providing authentic tasks, encouraging constructive controversy, asking “why” questions, or encouraging student autonomy. 

Explanation: Promote the development of critical thinking by articulating or explicitly stating what is at stake, what strategies can be used, and what criteria must be met. This explanation can take the form of oral or written communication and should always be explicit and specific. Declaring and making things explicit can be accomplished in a variety of ways, including using critical thinking rubrics, developing elaborate concept maps, providing feedback on critical thinking, and engaging in discussion and reflection on critical issues.

This integrated approach, encompassing university-business collaboration and an educational protocol, underscores the significance of critical thinking in higher education. It provides a structured framework for nurturing this essential skill by aligning objectives, fostering partnerships, adapting curricula, and implementing ongoing evaluation practices. In doing so, educational institutions are better poised to equip students with the critical thinking skills needed to thrive in a rapidly evolving world.

## 6. Concluding Remarks or Can Critical THINKING Save the World?

In summary, the dynamic interaction between universities, businesses, and the evolving technology landscape, including the rise of artificial intelligence (AI) and online technologies, underscore the critical need to nurture and develop students’ critical thinking skills. As we navigate the challenges posed by AI and the ever-expanding digital realm, collaborative efforts between academia and industry have proven to be instrumental in preparing students for the future job market.

Incorporating real-world experiences, such as apprenticeships, into the curriculum is an important step toward improving students’ critical thinking skills in real-world contexts. Projects such as “Critical thinking across higher education curricula—CRITHINKEDU” and “Critical thinking for successful jobs—THINK4JOBS” have demonstrated the potential of these collaborations to bridge the gap between classroom learning and industry needs. In addition, the development of flexible curricula that can adapt to the evolving needs of the job market, especially considering online technologies, is essential. By integrating real-world challenges and case studies into the curriculum, students gain valuable problem-solving skills and are better prepared to navigate the complexities of the digital age.

Ongoing assessment and evaluation are critical components of this collaborative effort, ensuring that critical thinking remains a central focus and that students are making meaningful progress in acquiring this essential skill.

With the disruption of AI and the ubiquity of online technologies, the integration of critical thinking into higher education curricula is more important than ever. It enables students not only to thrive in a technology-driven world, but also to contribute to a rational, democratic, and globally interconnected society. The partnerships forged between universities and businesses, along with a well-defined educational protocol, provide a roadmap for cultivating these essential skills and preparing students for the challenges and opportunities of the future job market. The imperative to foster critical thinking in university curricula remains a fundamental step in equipping tomorrow’s workforce to navigate the complexities of an AI-influenced job market and a rapidly changing world. 

Lilienfeld (2007, para. 3) said it well: “The greatest threat to the world is ideological fanaticism, by ideological fanaticism I mean the unshakeable conviction that one’s belief system and that of other in-group members is always right and righteous and that others’ belief systems are always wrong and wrong-headed”. Imagine a world where (most or even many) people use the skills of critical thinking. Just maybe, CT could save the world.

The job market will require a psychologically adaptable toolkit, and we propose that critical thinking is an essential component therein. The disruptions imposed by new technological advances such as AI will require students to learn new employable skills because we will need not just an engineer, but a critical thinking engineer; not just a programmer, but a critical thinking programmer; and not just a journalist, but a critical thinking journalist. The dignity of workers—their humanity and our collective survival—may well depend on CT, a very human creation.

## Figures and Tables

**Table 1:** Sample items forming Critical Thinking Self-Assessment Scale (CTSAS), Nair (2011).

NO. of Item	Item	Skill
1	*I try to figure out the content of the problem.*	Interpretation
10	*I examine the similarities and differences among the opinions posed for a given problem.*	Analysis
22	*I seek the accuracy of the evidence supporting a given judgment.*	Evaluation
31	*I figure out alternate hypotheses/questions, when I need to solve a problem.*	Inference

**Table 2:** Sample items from Student-Educator Negotiated Critical Thinking Dispositions Scale (SENCTDS), developed by Quinn et al. (2020).

No. of Item	Item	Disposition
2	*When faced with a decision, I seek as much information as possible.*	Reflection
6	*I often miss out on important information because I’m thinking of other things.*	Attentiveness
11	*I know what I think and believe so it’s not important to dwell on it any further.*	Open-mindedness
13	*I take notes so I can organize my thoughts.*	Organization
21	*Even if material is difficult to comprehend, I enjoy dealing with information that arouses my curiosity.*	Intrinsic goal motivation
